# Cloning and identification of the CTLA-4IgV gene and functional application of vaccine in Xinjiang sheep

**DOI:** 10.1515/biol-2022-0524

**Published:** 2022-11-23

**Authors:** Huifang Kong, Shangqi Zhao, Jia Zheng, Bin Liu, Yanxia Zhou, Yanmin Li, Wentao Zhou, Xiaotao Zhou

**Affiliations:** Department of Immunology, Basic Medical College, Xinjiang Medical University, Urumqi, China; Department of Immunology, College of Basic Medicine of Xinjiang Medical University, Urumqi, China; Department of Clinical Laboratory, The First Affiliated Hospital of Xinjiang Medical University, Urumqi, China; Department of Respiratory Medicine, The Fifth Affiliated Hospital of Xinjiang Medical University, Urumqi, China

**Keywords:** Xinjiang sheep, CTLA-4 extracellular domain, dendritic cells, Bioinformatics

## Abstract

Cytotoxic T lymphocyte-associated antigen 4 (CTLA-4) is an important surface molecule of activated T cells that has a strong affinity with the B7 molecule on the surface of antigen-presenting cells. Among these molecules, the CTLA-4 extracellular region (CTLA-4 IgV) may be used as a novel immune adjuvant molecule for delivering antigens and inducing strong humoral and cellular immune responses. In this study, bioinformatics analysis was performed to determine and clone the extracellular region of Xinjiang sheep CTLA-4 (NM_001009214). The CTLA-4 IgV gene was amplified and ligated into the pMD19-T vector, and the positive bacteria were screened by blue-white spots for sequencing and comparison. The correctly sequenced CTLA-4 IgV was digested and then ligated into the prokaryotic expression vector pET-30a(+). The plasmid pET30a–CTLA-4 IgV was constructed to induce the expression of the recombinant protein CTLA-4 IgV. Thereafter, CTLA-4 IgV was identified. Clustal X multiple sequence alignment revealed that the protein sequence of Xinjiang sheep CTLA-4 IgV was different from that of the known CTLA-4 extracellular region. The 3D protein structure of Xinjiang sheep CTLA-4 IgV was constructed via the bioinformatics method. Subsequently, molecular docking between the Xinjiang sheep CTLA-4 IgV protein and the B7 molecule was conducted. Results revealed multiple binding sites in the extracellular region of Xinjiang sheep CTLA-4, and two multiple interactions ensured stable binding after docking. The functionality of the Xinjiang sheep CTLA-4 IgV protein was further verified by fusing the CTLA-4 extracellular V region with EgG1Y162, a protective protein from *Echinococcus granulosa*, and the purified recombinant protein CTLA-4 IgV–EgG1Y162 was expressed with the mouse bone marrow-derived. The addition of the Xinjiang sheep CTLA-4 IgV protein at the amino terminus promoted the binding of EgG1Y162 to dendritic cells (DCs) and increased the maturation rate of these cells, further indicating that the protein could effectively improve the antigen presentation ability of DCs. The CTLA-4 extracellular domain protein of Xinjiang sheep is unique and has the potential to promote the presentation of the fusion protein by DCs as an adjuvant. The cloning and expression of this gene provide new measures and ideas for the preparation of the Xinjiang sheep vaccine to prevent zoonotic diseases.

## Graphical abstract

**Figure:**
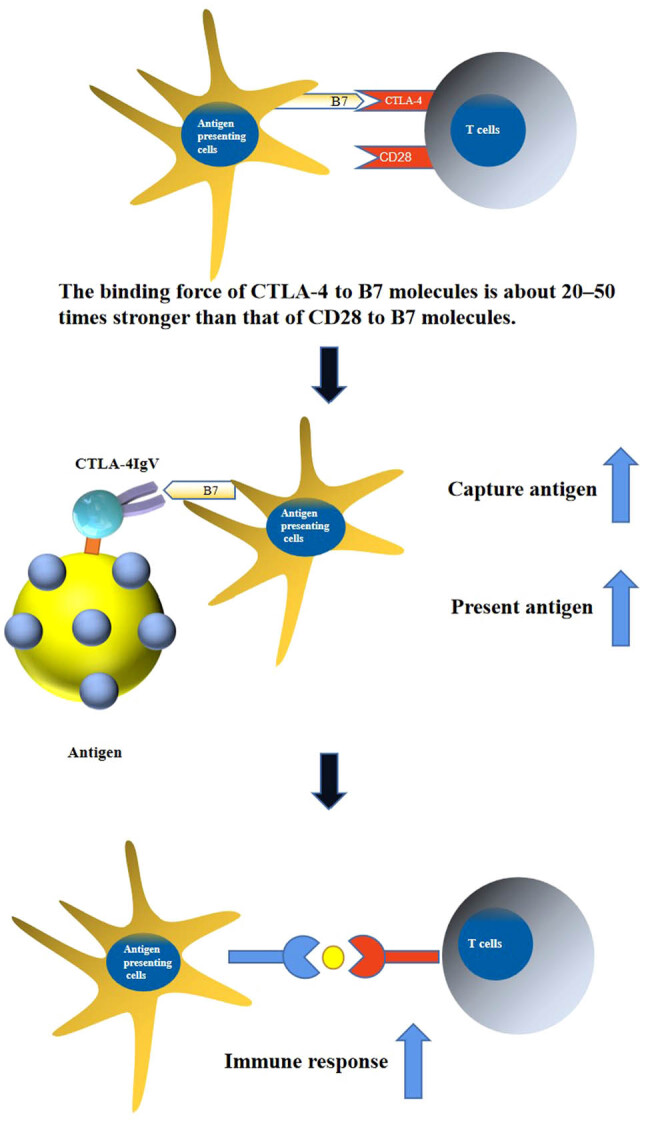

## 1 Introduction

Hydatid disease, also known as echinococcosis, is a parasitic disease that seriously endangers human and animal health [1]. Xinjiang Province is home to a large industry of animal husbandry. In this area, sheep are the intermediate host of *Echinococcus* and the main source of infection for the spread of hydatid disease. The transmission of hydatid disease can be effectively controlled by severing the life chain of *Echinococcus* in the intermediate host through vaccination. Immunizing animals with antigens that are fused to the extracellular domain of cytotoxic T lymphocyte-associated antigen 4 (CTLA-4) reportedly induces strong humoral and cellular immune responses. CTLA-4 is a member of the immunoglobulin superfamily, which is mainly expressed in activated lymphocytes and is a ligand-protein molecule involved in immune signal transmission. The monomer molecule of CTLA-4 consists of a 124-amino acid IgV extracellular region, a 26-amino acid transmembrane region, and a 36-amino acid cytoplasmic tail region. The extracellular region of IgV contains a hexapeptide motif (MYPPPY) binding the B7 molecule, which can bind the B7 molecule onto antigen-presenting cells [2]. The extracellular region of CTLA-4 alone cannot transmit immune signals into the intracellular region due to the lack of a transmembrane region and an intracellular region; thus, it does not have a negative immune regulation function. Although CTLA-4 and CD28 share 30% homology at the amino acid level, both proteins can specifically bind to B7 molecules [3]. However, the binding force of CTLA-4 to B7 molecules is about 20–50 times stronger than that of CD28 to B7 molecules. As a result of the high affinity of CTLA-4 and B7 molecules, specific antigens can be presented to APCs to improve the level of the immune response through the targeting and guiding effects of CTLA-4 [4]. In this study, the CTLA-4 IgV gene of Xinjiang sheep was cloned, and the prokaryotic expression plasmid pET30a–CTLA-4 IgV was constructed. The prokaryotic expression, purification, and preliminary verification of the functions of the CTLA-4 IgV protein elucidated the ideal conditions and offered new ideas for the later development of the Xinjiang sheep recombinant vaccine.

## 2 Materials and methods

### 2.1 Materials

#### 2.1.1 Experimental animals, plasmids, and strains

Sheep were provided by the Animal Center of Xinjiang Medical University. pMD19-T Vector and T4 DNA ligase were purchased from TakaRa Company. The prokaryotic expression vector pET30a was bought from Biovector Science Lab. Competent cells of *Escherichia coli* DH5a were prepared in our laboratory following conventional methods.

### 2.2 Primers

PCR primers were designed according to the sequence of the sheep CTLA-4 gene (AF02740) in GenBank. BamHI and KpnI restriction sites (crossed part) were introduced into positive and reverse primers, respectively. The primer sequences are as follows:

CTLA4-V F: 5ʹ-ACGGATCCTAATGTGACCCAGCCTCCAG-3ʹ

CTLA4-V R: 5 ʹ-TAGGTACCATCAGAATCCGGGCATGGTTC-3ʹ

### 2.3 Main reagents

The kit for separating sheep peripheral blood lymphocytes was acquired from Beijing Solaibao Technology Co., Ltd. Trizol and RNA reverse transcription kit were procured from Invitrogen Company. ConA was obtained from Sigma Company. The kit for extracting plasmids was purchased from Tiangen Company. Glue cutting recovery kit, PrimeSTAR HS (Primix), KpnI, BamHI, and BglII were bought from TakaRa Company. The other reagents used were analytically pure and procured from local suppliers.


**Ethical approval:** The research related to animal use has been complied with all the relevant national regulations and institutional policies for the care and use of animals. Specific pathogen-free C57 mice (6–8 weeks, 20 ± 2 g) were approved by Experimental Animal Center of Xinjiang Medical University (License No: SCXK(Xin)2016-0003).

## 3 Methods

### 3.1 Cloning of the CTLA-4 IgV gene

Total RNA of sheep lymphocytes was extracted by the Trizol method. cDNA was synthesized using Invitrogen reverse transcription kit according to the manufacturer’s instructions. The PCR procedure involved 94°C for 5 min, 94°C for 30 s, 60°C for 30 s, 72°C for 45 s for 35 cycles, and 72°C for 10 min. The DNA fragment recovered from gel cutting was inserted into the pMD19-T vector, and the recombinant plasmid pMD19-T–CTLA-4 IgV cultured overnight was transformed into susceptible cells. A single white colony was selected by screening blue and white spots. Finally, the recombinant plasmid pMD19-T–CTLA-4 IgV was digested using the restriction enzymes BamHI and KpnI and then sent to Quinterui Biotechnology Co., Ltd. for sequencing.

### 3.2 Construction of the prokaryotic expression plasmid pET30a–CTLA-4IgV

The recombinant plasmid pMD19-T–CTLA-4 IgV was sequenced using the restriction enzymes BamHI and KpnI, and the CTLA-4IgV gene fragment was recovered by gel cutting. According to the characteristics of BamHI and BglII as the cocktail enzymes, the recovered CTLA-4IgV was connected to the linear vector of pET30a digested by BglII and KpnI, transformed into receptive cells, and cultured overnight. Single colonies were randomly selected and then added into the liquid medium containing Amp. Plasmids were extracted and identified via PCR. The recombinant plasmid with the correct identification was sent to Quinterui Biotechnology Co., Ltd. for sequencing.

### 3.3 Induction expression and identification of the CTLA-4IgV protein

The prokaryotic expression plasmid pET30a–CTLA-4IgV was transformed into the host strain *E. coli* BL21 (DE3). A single clone was picked, inoculated in LB medium containing kanamycin, and cultured overnight with shaking. The proportion of the bacterial liquid containing the pET30a–CTLA-4IgV recombinant plasmid was 1:50. It was inoculated in LB liquid medium containing kanamycin, shaken, and cultivated until the optical density of the bacterial liquid ranged from 0.6 to 0.8. The bacterial liquid was placed into sterilized centrifuge tubes. The tubes were grouped (Table 1), and the cells were collected after induction and expression in accordance with the corresponding conditions. About 100 μL of precooled PBS was added to suspend the cells, and PMSF was added with a final concentration of 1 mmol/L. Thereafter, the cells were subjected to ultrasonic lysis twice. The cells were centrifuged at 12,000 rpm for 10 min at 4°C to separate the supernatant and pellet after induction. Subsequently, 4× protein loading buffer was added, boiled at 100°C for 10 min, and frozen at −20°C for future use. The induced expression of proteins was analyzed via sodium dodecyl sulfate-polyacrylamide gel electrophoresis (SDS-PAGE). The bacterial supernatant was electrophoresed by SDS-PAGE, electrotransferred from the gel onto a polyvinylidene fluoride membrane, blocked with 5% nonfat dry milk at room temperature, washed with 1× TBST, and added with mouse anti-His-Tag monoclonal antibody (1:2,000 dilution). The bacterial supernatant was incubated overnight at 4°C on a shaker, washed with 1× TBST, added with HRP rabbit anti-mouse antibody (1:3,000 dilution), and incubated at room temperature for 2 h. The supernatant was washed three times with 1× TBST for 15 min each time, added with ECL for color development, and observed.

**Table 1:** Recombinant protein expression groups

Induced condition IPTG	0.2 mmol/L	0.5 mmol/L	0.8 mmol/L
Not induced	1	5	9
28°C for 4 h	2	6	10
28°C for 2 h; 37°C for 2 h	3	7	11
37°C for 4 h	4	8	12

### 3.4 Sequence alignment results

All sheep CTLA-4 IgV protein sequences (AAD04380.1, P001009214.1, and XP027819684.1) registered in NCBI were downloaded. Clustal X was used to perform multiple sequence alignment of the V regions of different sheep CTLA-4 sequences.

### 3.5 Protein tertiary structure modeling

Using the online software SWISS-MODEL (https://swissmodel.expasy.org/) and I-TASSER (http://zhanglab.ccmb.med.umich.edu/I-TASSER/), [5] three hierarchical protein structures were predicted by homology modeling and threading and locally adjusted by spdbv software [6]. The tertiary structure display of the predicted protein was modeled using Chimera 1.6.2 software.

### 3.6 Molecular docking

CD80/B7 and CTLA-4IgV target protein structures were obtained using the online server SWISS-MODEL homology modeling (https://swissmodel.expasy.org/, homologous modeling: 1I8L, 7ELX). All protein structures were processed in the molecular operating environment platform, including the removal of water and ions, protonation, addition of missing atoms and completion of missing groups, and protein energy minimization. Using HDOCK software, the protein was set to rigid, the docking contact site was set to the full surface, the conformation generated after docking was set to 100, and the scoring function was used to select the conformation with the most negative energy.

### 3.7 *In vitro* experiments to detect the binding ability of recombinant proteins to mouse-derived dendritic cells

Six- to eight-month-old C57 mice were sacrificed by cervical dislocation. The tibiae and fibulae of the mice were obtained under aseptic conditions. Both ends of the bones were severed and washed with PBS buffer. The bone marrow was pushed out until the bones turned white. The cells were strained through a sterile 200-mesh strainer to remove tissue debris. Bone marrow hematopoietic stem cells were collected, made into a cell suspension, and plated. The stimulators rmGM-CSF and rmIL-4 were added, and dendritic cells (DCs) were obtained when the cells were cultured until the 7th day. The cells were harvested after 24 h of stimulation with purified EgG1Y162 and HIS–CTLA-4 IgV–EgG1Y162. About 50 μL of PBS containing 1 μL of anti-His-Tag antibody was added to the tube, which was incubated at 4°C for 30 min in the dark. The expression of antigen molecules bound onto the surface of DCs by the target protein was detected within 4 h.

### 3.8 *In vitro* experiments to detect the ability of recombinant proteins to promote DC maturation

DCs were collected after incubating them with the HIS–CTLA-4 IgV–EgG1Y162 protein for 24 h *in vitro*. About 1 × 10^6^ cells were placed in each flow tube. After mixing the cells, PBS containing 1 μL of CD86^+^, 1 μL of CD11c^+^, 1 μL of CD45^+,^ and 1 μL of I-Ab antibody was added. After incubation at 4°C for 30 min in the dark, 300 μL of 1× PBS was added to resuspend the cells. The percentage of mature DCs (mDCs) was detected within 4 h.

## 4 Results

### 4.1 Cloning of CTLA-4 IgV gene of Xinjiang sheep

**Figure 1:**
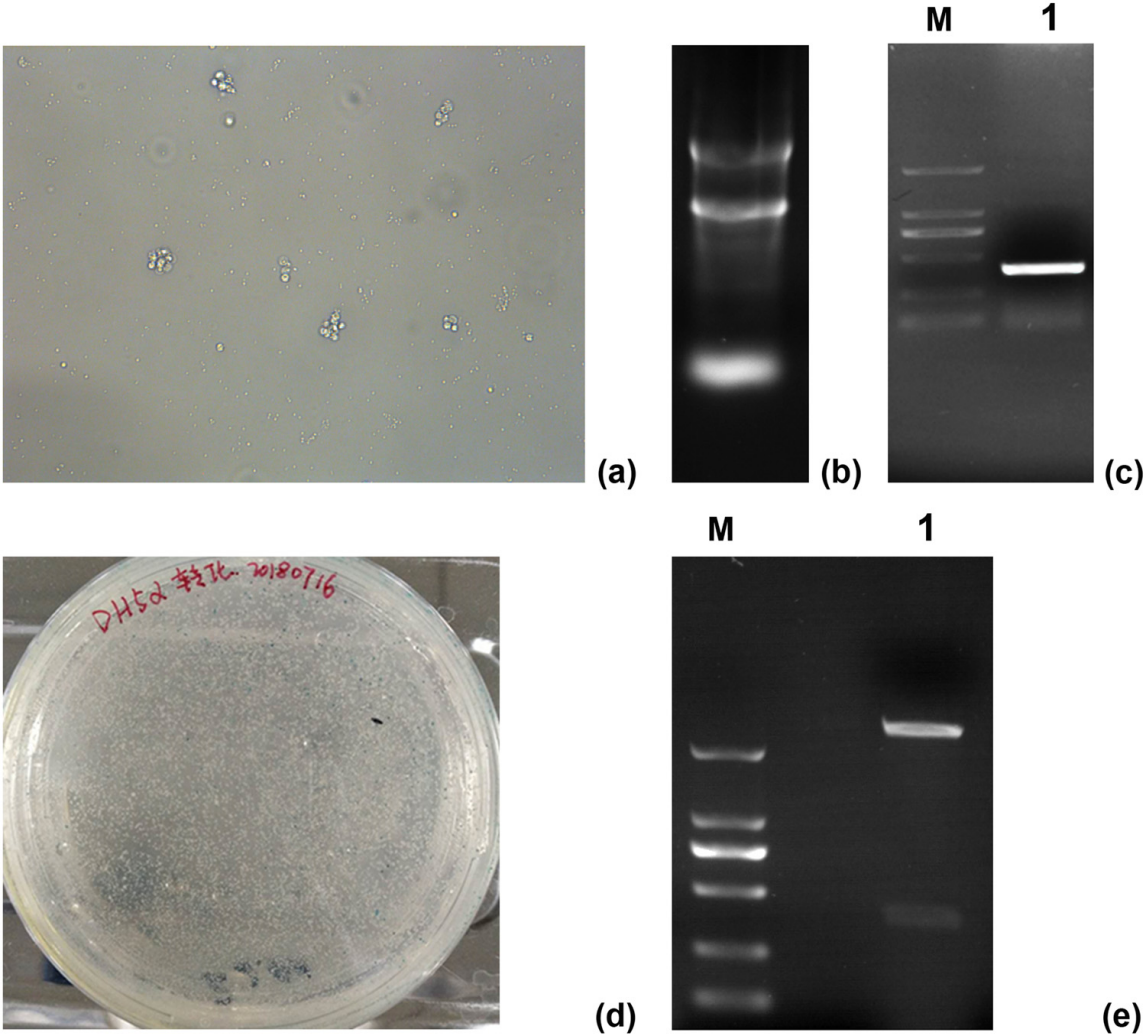
Cloning and identification of the CTLA-4 IgV gene: (a) Con A stimulated lymphocytes (200×); (b): electrophoresis of total RNA; (c) plasmid PCR gel electrophoresis, M: 2,000 bp DNA marker, 1: PCR target band; (d) constructed CTLA-4 IgV gene fragment PMD19-T was transformed into competent DH5a cells, and positive clones were screened by blue and white spots; (e) plasmid BamHI and KpnI gel electrophoresis after double digestion, M: 2,000 bp DNA marker, 1: Plasmid electrophoresis was performed after double enzyme digestion.

After Con A induction for 48 h, the volume of sheep peripheral blood lymphocytes increased, the nuclei showed an obvious mitotic phase, and the cells proliferated and formed clones (Figure 1a). Total RNA extracted from the sheep lymphocytes was determined using a nucleic acid quantitative analyzer. The OD_260/280_ ratio was 1.85, and the extracted RNA concentration was 163.7 ng/μL. Three bands of 5 S, 18 S, and 28 S RNA were observed using 1% agarose gel electrophoresis (Figure 1b). The PCR product of the CTLA-4IgV gene was detected using 1% agarose gel electrophoresis. The specific amplification band of 369 bp was detected between 250 and 500 bp, and the fragment size was consistent with the expected size (Figure 1c). The primer dimer at 100 bp did not affect the subsequent experiments. The CTLA-4 IgV gene was successfully inserted into the pMD19-T vector. The successfully transformed colonies were white, whereas the colonies without gene insertion were blue (Figure 1d). Three white single colonies were selected for amplification, and a small plasmid extraction kit was used for plasmid extraction. After BamHI and KpnI double digestion, two fragments with the size of 2,600 and 369 bp were detected using 1% agarose gel electrophoresis (Figure 1e). The recombinant plasmid was sent to Quintairui Biotechnology Co., Ltd. for sequencing. The sequencing results were compared with those found in NCBI BLAST. The comparison revealed 99.9% homology with the ctLA-4 extracellular region of sheep. The recombinant plasmid pMD19-T–CTLA-4 IgV was successfully constructed, and the amplified fragment was the sequence of the sheep CTLA-4 IgV gene.

### 4.2 Construction and Identification of prokaryotic expression plasmid pET30a–CTLA-4 IgV 

**Figure 2:**
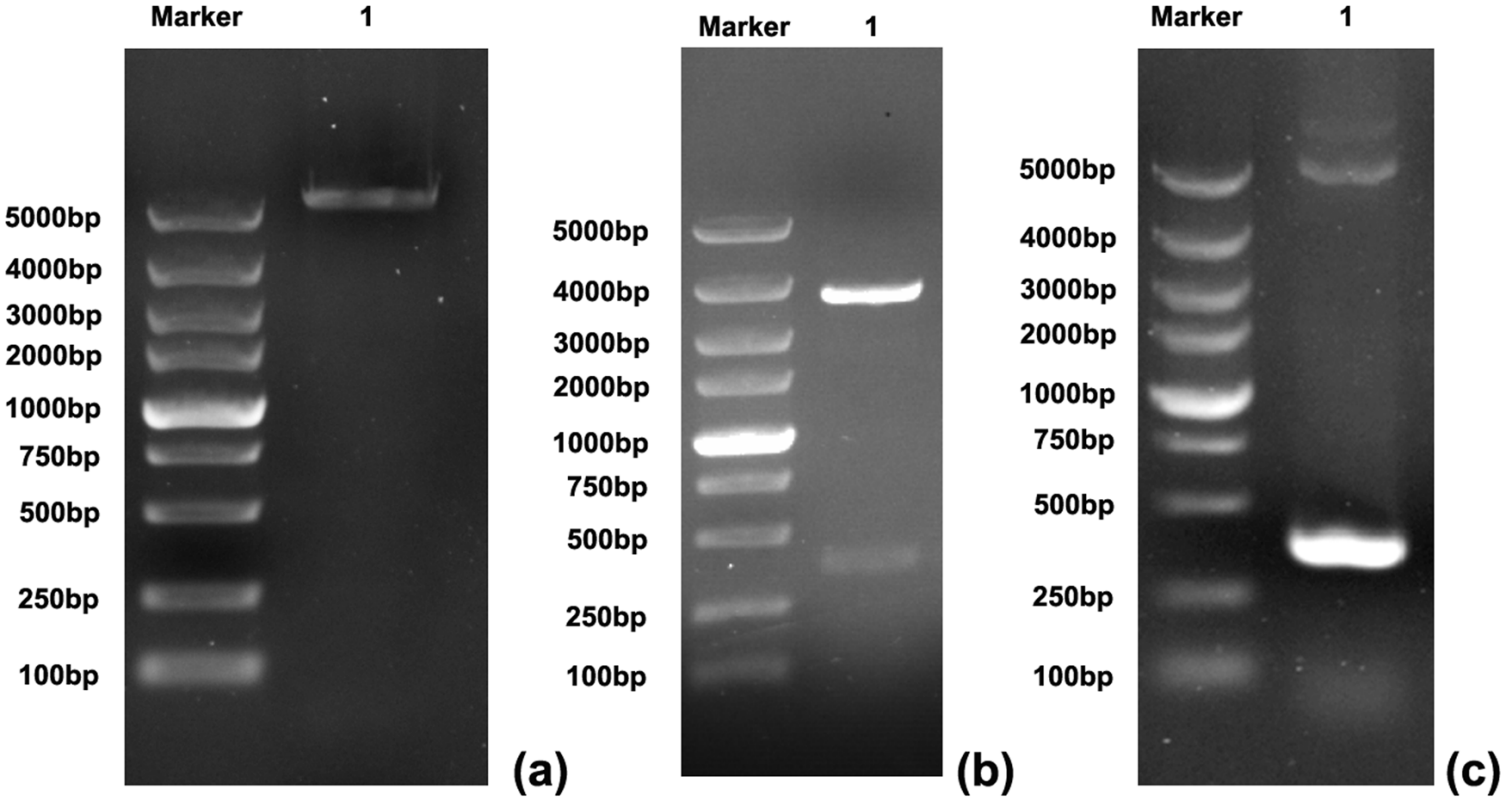
Identification of the recombinant plasmid pET30a–CTLA-4 IgV: (a) pET30a gel electrophoresis after double enzyme digestion, M: DNA marker 5000 (bp), 1: target band after plasmid enzyme digestion; (b) pMD19-T–CTLA-4 IgV double digestion electrophoresis, M: DNA marker 5000 (BP), 1: target band after plasmid digestion; (c) plasmid PCR gel electrophoresis, M: DNA marker 5000 (BP) 1: PCR target band.

The prokaryotic expression vector pET30a was digested by BglII and KpnI, and a linear pET30a (Figure 2a) was obtained by gel electrophoresis. The plasmid pMD19-T–CTLA-4 IgV was digested by BamH I and KpnI, and a linear CTLA-4 IgV fragment (Figure 2b) was obtained at 369 bp by gel electrophoresis. The plasmid pET30a–CTLA-4 IgV was constructed by connecting the CTLA-4 IgV fragment and the linear pET30a band. The constructed plasmid pET30a–CTLA-4 IgV was transformed into competent *E. coli* DH5a cells, and single colonies were selected. These colonies were added into 5 mL of liquid medium containing 100 g/mL ampicillin. After plasmid DNA was extracted, the recombinant plasmid was used as the template and amplified by PCR. The results of 1% agarose gel electrophoresis showed that the amplified product was about 360 bp, which was consistent with the size of the expected gene fragment (Figure 2c). The recombinant plasmid pET30a–CTLA-4 IgV was sequenced, and the sequencing result was consistent with the gene sequence published in GenBank. After sequencing verification, the recombinant plasmid pET30a–CTLA-4 IgV was successfully constructed, and its clone was confirmed by sequencing.

### 4.3 Induction and identification of CTLA-4IgV protein

**Figure 3:**
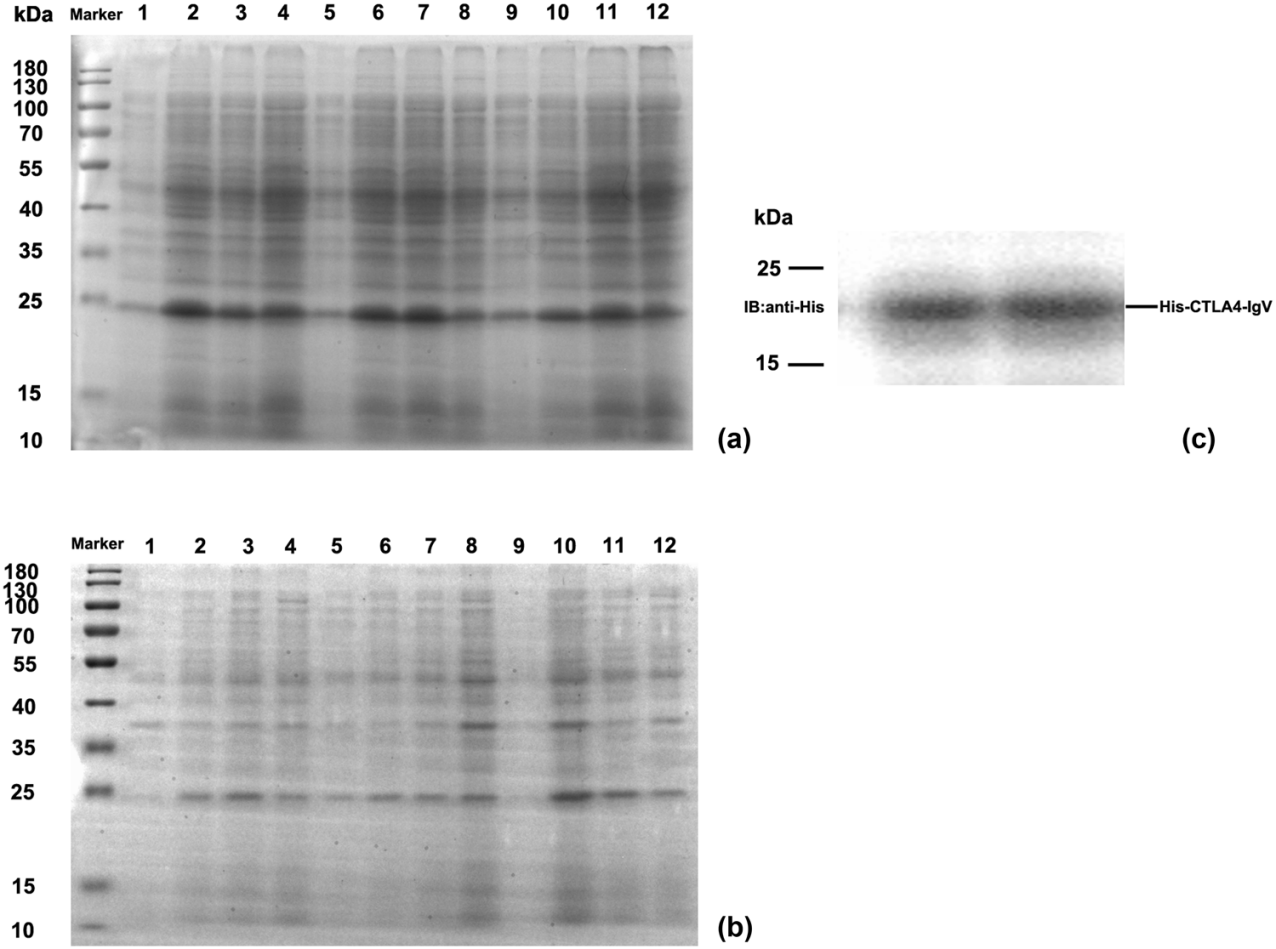
SDS-PAGE and Western blot analysis of the CTLA-4 IgV recombinant protein: (a) SDS-PAGE analysis of the recombinant protein CTLA-4 IgV in the supernatant; M: Protein molecular mass standards 1–4 are pET30a–CTLA-4 IgV induced at the IPTG concentration of 0.2 mmol/L for 0 h, at 28°C for 4 h, at 28°C for 2 h plus 37°C for 2 h, and at 37°C for 4 h, respectively. Protein molecular mass standards 5–8 are pET30a–CTLA-4 IgV induced at the IPTG concentration of 0.5 mmol/L for 0 h, 28°C for 4 h, 28°C for 2 h plus 37°C for 2 h, and 37°C for 4 h, respectively. Protein molecular mass standards 9–12 are pET30a–CTLA-4 IgV induced at the IPTG concentration of 0.8 mmol/L for 0 h, at 28°C for 4 h, at 28°C for 2 h plus 37°C for 2 h, and at 37°C for 4 h, respectively. (b) SDS-PAGE analysis of the recombinant protein CTLA-4 IgV in the precipitate; M: Protein molecular mass standards 1–4 are pET30a–CTLA-4 IgV induced at the IPTG concentration of 0.2 mmol/L for 0 h, at 28°C for 4 h, at 28°C for 2 h plus 37°C for 2 h, and at 37°C for 4 h, respectively. Protein molecular mass standards 5–8 are pET30a–CTLA-4 IgV induced at the IPTG concentration of 0.5 mmol/L for 0 h, at 28°C for 4 h, at 28°C for 2 h plus 37°C for 2 h, and at 37°C for 4 h, respectively. Protein molecular mass standards 9–12 are pET30a–CTLA-4 IgV induced at the IPTG concentration of 0.8 mmol/L for 0 h, 28°C for 4 h, 28°C for 2 h plus 37°C for 2 h, and 37°C for 4 h, respectively. (c) Western blot analysis of CTLA-4IgV recombinant protein; M: Protein molecular quality standard; 1–2: CTLA-4 IgV protein.

pET30a–CTLA-4IgV was transformed into *E. coli* BL21 (DE3)-competent cells, induced by IPTG with different concentrations and time. The supernatant and precipitate were identified by SDS-PAGE electrophoresis. Figure 3a shows that the CTLA-4IgV protein had an expression band at about 21 kDa, which was consistent with expectations, and the protein expression level in the supernatant was substantially higher than that in the precipitation. At 28°C, the IPTG concentration was 0.2 mmol/L, and the induced expression of the recombinant protein was the highest in the supernatant after 4 h of induction. Western blot analysis was performed to identify the recombinant CTLA-4 IgV protein. As shown in Figure 3c, the induced bacteria showed obvious bands at 21 kDa, which were consistent with the specific band locations expressed in SDS-PAGE electrophoresis, indicating that the recombinant CTLA-4 IgV protein was successfully expressed.

### 4.4 Multiple sequence alignment of the Xinjiang sheep CTLA-4 IgV protein and construction of the molecular evolution tree

**Figure 4:**
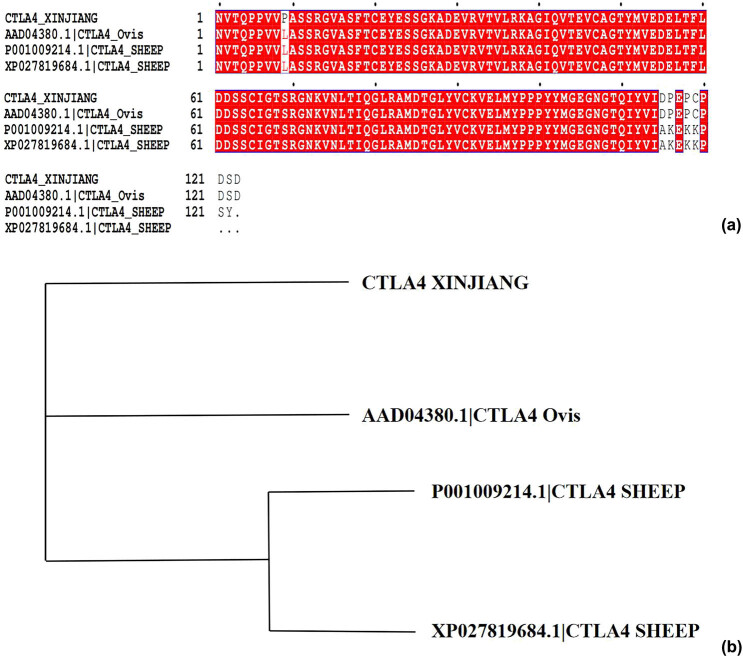
Multiple sequence alignments and molecular evolutionary trees: (a) multiple sequence alignment of Xinjiang sheep CTLA-4 IgV via Clustal X software; (b) molecular evolutionary trees.

All sequences of the sheep CTLA-4 IgV protein registered in NCBI (AAD04380.1, P001009214.1, and XP027819684.1) were downloaded. These sequences and those of our cloned Xinjiang sheep CTLA-4 IgV protein (CTLA-4IgV XINJIANG) were subjected to multiple sequence alignments via Clustal X software. As shown in Figure 4a, the sequences of the CTLA-4 IgV protein of Xinjiang sheep are unique in some aspects. For instance, the 9th amino acid is P, which also indicates the diversity of species. As can be observed from the molecular evolutionary tree (Figure 4b), the sequences of the CTLA-4 IgV protein of Xinjiang sheep were relatively independent of one another.

### 4.5 Tertiary structure modeling of the Xinjiang sheep CTLA-4 IgV protein by bioinformatics analysis

**Figure 5:**
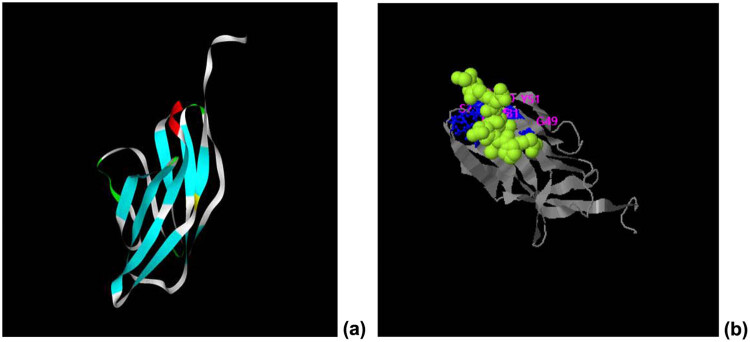
Ligand binding pattern of CTLA-4 IgV. (a) Prediction of the tertiary structure of the His–CTLA-4 IgV protein. (b) Possible ligand binding site of the recombinant protein CTLA-4 IgV.

The structure of the recombinant protein CTLA-4 IgV was predicted by the I-TASSER online server (Figure 5a). C-scores typically range [−5, 2], with high scores indicating high confidence in a model. TM-score and RMSD score are necessary indicators to predict the quality of modeling. TM-score is a measure of similarity between two structures. The recombinant protein was mainly composed of a β lamellar structure (*C*-score = −0.03, estimated TM-score = 0.71 ± 0.12, and estimated RMSD = 4.4 ± 2.9A). Moreover, the possible ligand binding sites of ctLA-4 IgV were predicted to be located at amino acid residues at positions 25–26, 29–31, 49–51, and 95–96 (*C*-score = 0.16; Figure 5b).

### 4.6 Interaction analysis and docking results of CD80/B7 and CTLA-4 IgV

**Figure 6:**
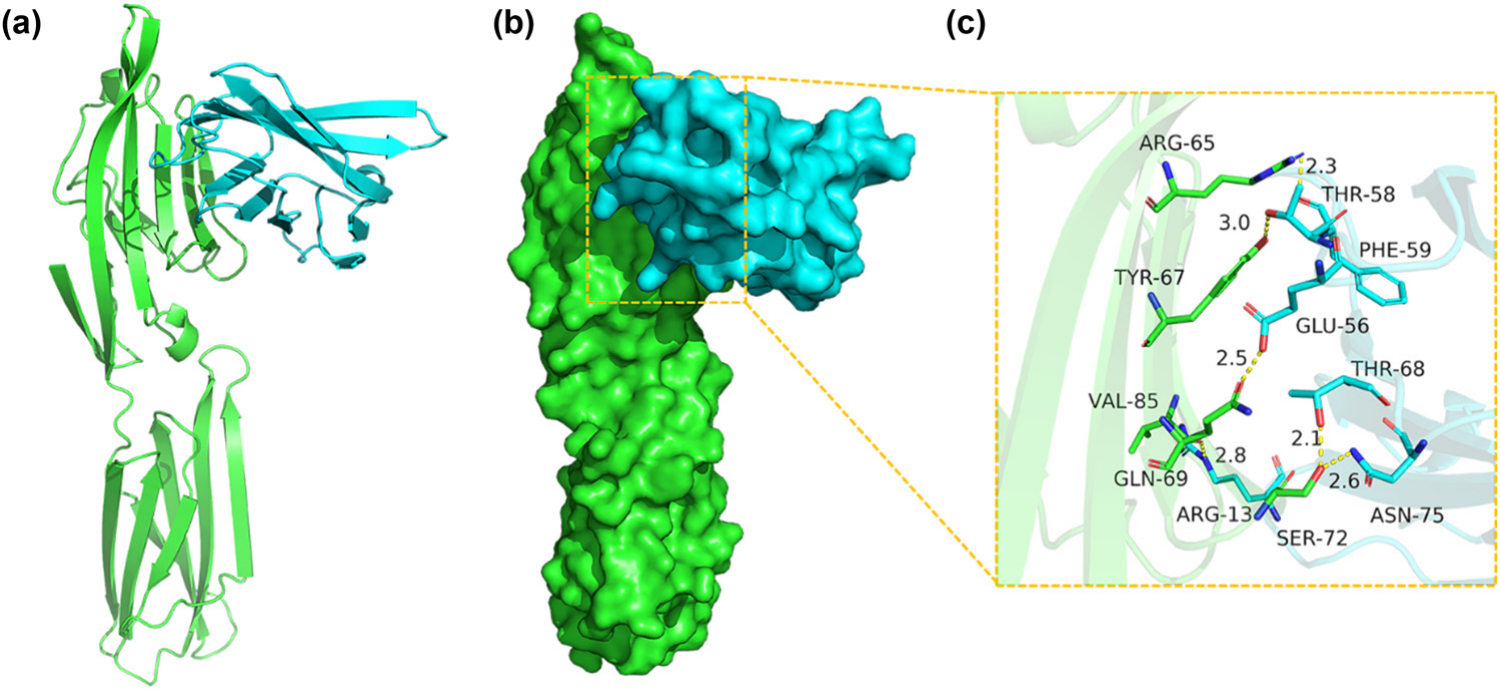
Binding mode of the CD80/B7 complex (green) with CTLA-4 IgV (blue). (a) The 3D structure of the complex. (b) Rendering of the surfaces of CD80/B7 and the CTLA-4 IgV protein. (c) Detailed binding mode of CD80/B7 with CTLA-4 IgV. Yellow dash represents a hydrogen bond or a salt bridge.

The binding score of CD80/B7 to CTLA-4 IgV protein was 268.50 kcal/mol. The binding site package of CD80/B7 protein was as follows. The binding sites of VAL-85, ARG-65, TYR-67, GLN-69, SER-72, and other amino acid residues included ARG-13, PHE-59, THR-58, GLU-56, THR-68, ASN-75, and other amino acid residues (Figure 6).

### 4.7 CTLA-4 IgV promotes the binding of EgG1Y162 to DCs

**Figure 7:**
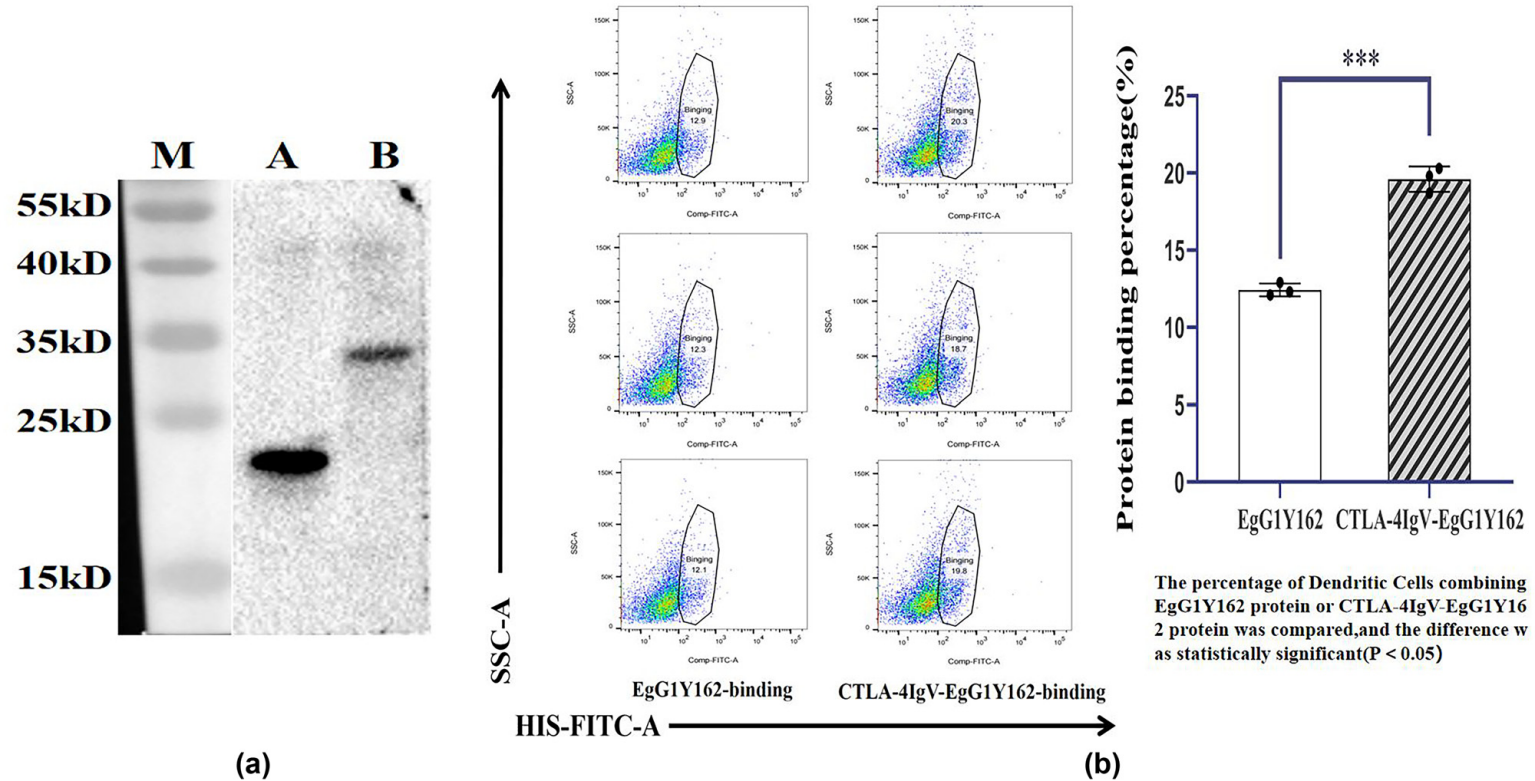
Binding ability of the purified protein CTLA-4 IgV–EgG1Y162 to dendritic cells: (a) purified recombinant proteins EgG1Y162 and CTLA-4 IgV–EgG1Y162 were identified by Western blot; M: Protein molecular quality standard; A: Purified EgG1Y162 protein; (b) purified CTLA-4 IgV–EgG1Y162 protein. (b) Binding capacity of antigen molecules to dendritic cells as determined via flow cytometry.

**Figure 8:**
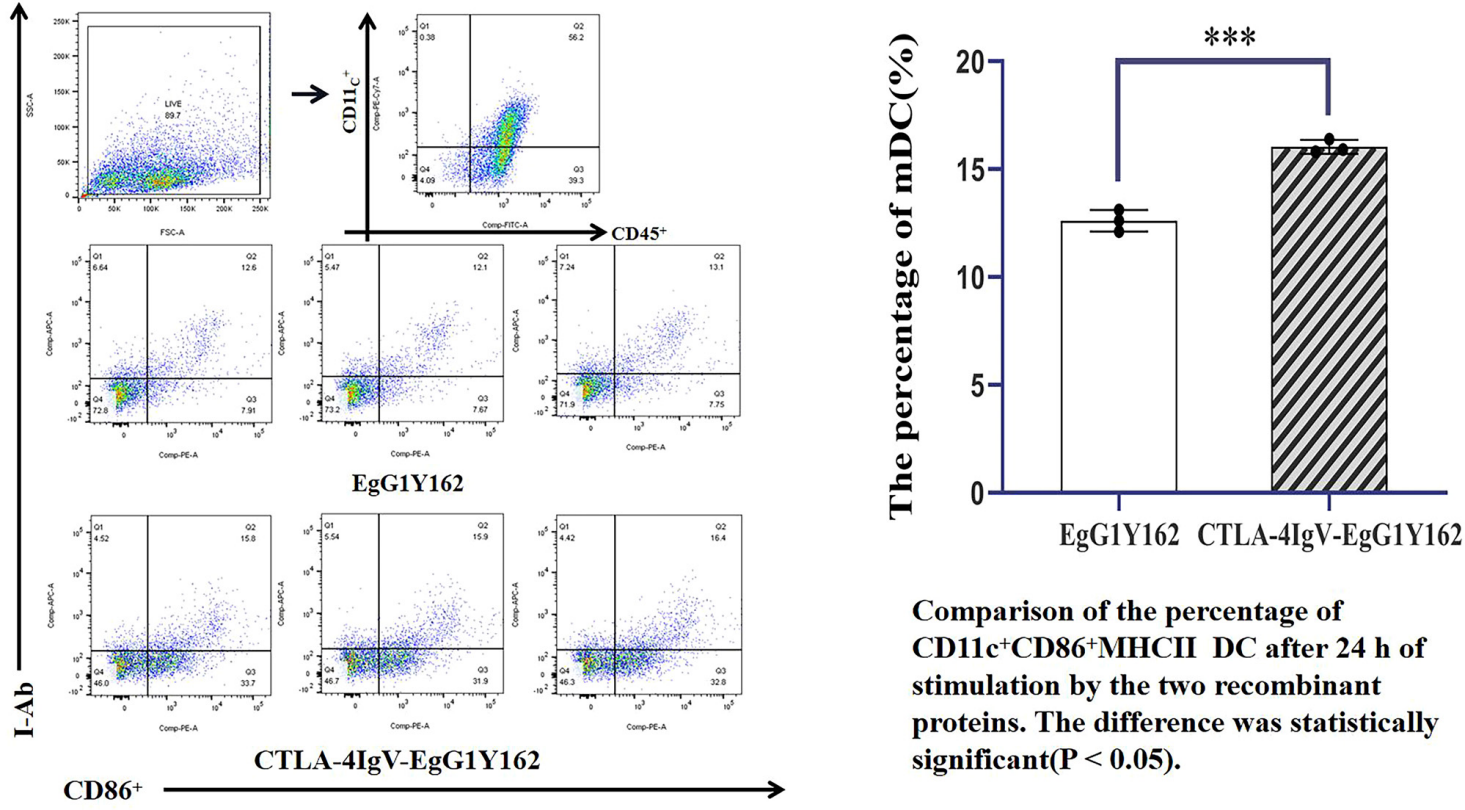
Ability of antigen molecules to promote dendritic cell maturation as determined via flow cytometry.

The purified proteins EgG1Y162 and CTLA-4IgV–EgG1Y162 were identified by His antibody. Western blot analysis revealed that protein EgG1Y162 and protein CTLA-4 IgV–EgG1Y162 displayed obvious bands at 20.5 and 29 kDa, respectively (Figure 7). Further experiments showed that the proportion of DCs bound to His–CTLA-4 IgV–EgG1Y162 (12.433 ± 0.4163%) was significantly higher than that of cells bound to His–EgG1Y162 after the DCs were co-cultured with the two purified recombinant proteins for 24 h (19.600 ± 0.8185%, *P* < 0.05; Figure 8).

#### 4.7.1 CTLA-4 IgV–EgG1Y162 can promote DC maturation *in vitro*

Flow cytometry was conducted to detect the expression of CD45-positive immune cells, DC surface marker CD11C^+^, and mDC surface markers CD86 and MHCII after antigen stimulation *in vitro*. The percentages of CD86 + and MHC-II phenotypes in CD45^+^ CD11c^+^ were 12.600 and 0.500, respectively, after 24 h of antigen stimulation with EgG1Y162 (Figure 8). After 24 h of stimulation with His–CTLA-4 IgV–EgG1Y162 antigen, the percentage of DC cell maturation was 16.033 ± 0.3215%. The percentage of mDC substantially increased, and the difference was statistically significant (*P* < 0.05).

## 5 Discussion

CTLA-4, a member of the B7/CD28 family that was first discovered by Brunet et al. [2], is a unique receptor on the surface of cytotoxic T lymphocytes. CTLA-4 shares structural similarities with CD28 [7] with 70% homology at the DNA level and 31% homology at the amino acid level. Both proteins are transmembrane receptors expressed on the surface of T cells, and they are collectively called the CD28 family of molecules [8]. B7 is an important costimulatory molecule that mediates T-cell activation. CD28 and CTLA-4 competitively bind to the common ligand B7, which inhibits T-cell proliferation and exerts a negative immunoregulatory effect [9]. T Cells are regulated to maintain the body’s immune tolerance [10]. However, the extracellular region of CTLA-4 alone lacks a transmembrane region and an intracellular region; the immune signal cannot be transmitted into the cell [11,12]. Thus, CTLA-4 does not have a negative regulatory effect. The binding force of CTLA-4 to B7 molecules is about 20–50 times stronger than that of CD28 to B7 molecules. Using this feature of CTLA-4 IgV can target and guide antigen presentation to APC cells.

Western China has the highest prevalence of cystic and alveolar echinococcosis. Sheep are the main intermediate host of these diseases, and some sheep farmers lack sufficient understanding of scientific breeding and pay little attention to the harm that parasites pose. Parasitic disease is a chronic wasting disease. If sheep are infected with parasites, their health and production performance are reduced, leading to a certain degree of economic loss. In Xinjiang Province where animal husbandry is the main industry, the health of sheep is crucial to animal husbandry production [13].

Therefore, we investigated the extracellular region of Xinjiang sheep CTLA-4. We constructed the prokaryotic expression plasmid pET30a–CTLA-4 IgV, introduced this vector into *E. coli* prokaryotic cells, induced its expression, and obtained the recombinant protein CTLA-4 IgV. The classic prokaryotic expression vector pET30a plasmid has a small tag His protein, which is composed of six histidines (His) that can minimally interfere with the structure of the expressed protein [14]. To harness the high affinity between CTLA-4 IgV and the antigen-presenting cell B7 molecule to prepare the recombinant vaccine fused with the CTLA-4 IgV protein at the amino-terminal (n-terminal), we attempted to construct the CTLA-4 IgV gene into the upstream of the MCS region of the plasmid when constructing the plasmid. We cloned the CTLA-4 IgV protein of Xinjiang sheep for gene sequencing and then translated it. We compared the multiple sequences of the CTLA-4 IgV protein with the existing sheep amino acid sequences in NCBI via Clustal X software. We observed differences in the 9th amino acid sequence of Xinjiang sheep, and these differences reflected the polymorphism of the Xinjiang sheep CTLA-4 gene in different species. Numerous studies have shown that CTLA-4 polymorphism is important in multiple fields of study because polymorphism leads to a large size, and large alleles are not conducive to stable mRNA transcription [15]. Given that the polymorphism of CTLA-4 is closely related to its function, the cloned CTLA-4 IgV protein with specific amino acids has been subjected to exploratory experiments [16]. Because of the limitations and short-comings of traditional vaccine development methods, high time cost, and low effectiveness, immunoinformatics approaches are being used by many scientists to develop a vaccine to prevent hydatidosis [17]. We fused CTLA-4 IgV with a potential vaccine candidate molecule, namely, EgG1Y162, which offers good protection against cystic hydatid disease. We observed the fusion protein CTLA-4 IgV–EgG1Y162 with DCs through *in vitro* experiments. The results demonstrated that DCs had a stronger binding ability to CTLA-4 IgV–EgG1Y162 than to EgG1Y162, and they were more likely to mature under protein stimulation. The results indicated that the fusion of CTLA-4 extracellular region with antigen could enhance the immunogenicity of antigen effectively [18].

The tertiary structure of the CTLA-4 IgV protein in Xinjiang sheep was obtained via I-TASSER online analysis software. PHYRE I-TASSER usually gives the first and most reliable template parameter. C-score is the confidence evaluation of the I-TASSER quality prediction model, and its calculation is based on the parameters of the simulated assembly in the sense of thread template alignment and structural alignment [19]. A protein TM-score of 0.71 shows a correct topology model and predicts that the protein ligand may be the binding site. In this study, molecular docking was also used to evaluate the advantages and disadvantages of protein interaction in real time according to the principles of geometric complementarity and energy complementation. Moreover, it was used to identify the best binding mode of two molecules. HDOCK software simulates the docking of Xinjiang sheep CTLA-4 IgV and CD80/B7. The contact residues of CD80/B7 and CTLA-4 IgV protein can form various interactions, such as a salt bridge, hydrogen bond, hydrophobic interactions, and other interactions. These existing interaction forces can effectively improve the stability of CD80/B7 and the CTLA-4 IgV protein complex, which shows that the extracellular region of Xinjiang sheep CTLA-4 can efficiently bind to CD80/B7 molecules.

Our study not only reported for the first time the unique amino acid sequence of the CTLA-4 IgV region of Xinjiang sheep, but it also confirmed through bioinformatics analysis and *in vitro* experiments that the fusion of the CTLA-4 molecule in the extracellular region of Xinjiang sheep at the amino-terminal could promote the binding of DCs to protein and ultimately promote the maturation of DC.

Thus, this region has a potential role in promoting immune response. This study provides insights into the ideal conditions for the development of a new Xinjiang sheep vaccine design. Such a vaccine design will hopefully ameliorate the high incidence of infectious diseases in sheep in Xinjiang and promote the sustainable high-quality and healthy development of animal husbandry in the province.

## Figures and Tables

**Figure A1:**
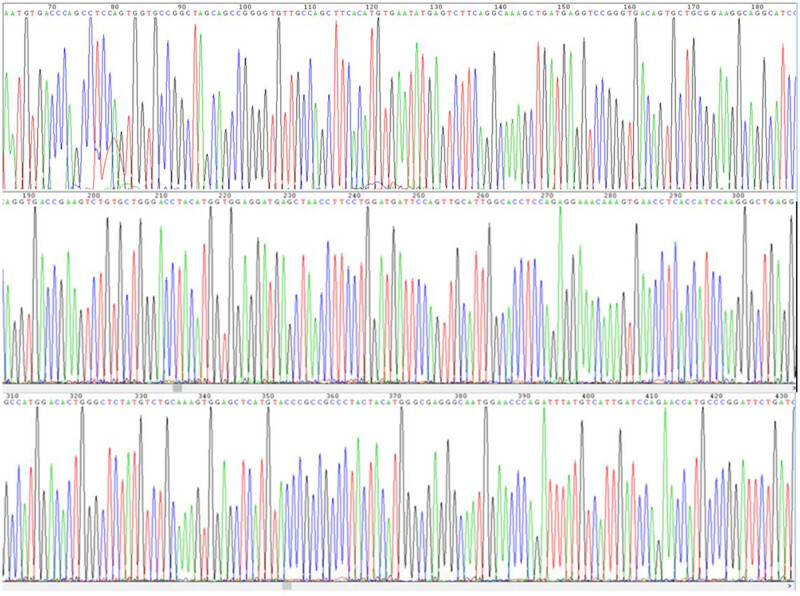
Sequencing results of the recombinant plasmid pET30a–CTLA-4 IgV.
